# Supplementary material for: Measurements of damage and repair of binary health attributes in aging mice and humans reveal that robustness and resilience decrease with age, operate over broad timescales, and are affected differently by interventions
Source: eLife. 2022 Nov 21;11:e77632. doi: 10.7554/eLife.77632 (PMC9725749; doi:10.7554/eLife.77632)
Supplement: Supplementary file 1. [file elife-77632-supp1.docx]

**SUPPLEMENTARY TABLE 1: EVIDENCE FOR DEFICITS THAT CAN REVERSE IN THE FRAILTY INDEX**

| **SYSTEM** | **DEFICIT** | **EVIDENCE THAT DEFICIT CAN REPAIR** | **REFERENCE** |
| --- | --- | --- | --- |
| **Integument** | Alopecia | Alopecia can be caused by overgrooming, fur nibbling and stress. This is reversible by removing a “Barbering” mouse and/or improved thermoregulation with extra nesting material. | Sarna et al., 2000; Wilkinson et al., 2020 |
|  | Loss of fur colour | Stress and aging lead to loss of fur colour in mice and this can be reversed by suppression of melanocyte stem cells proliferation. | Zhang et al., 2020 |
|  | Dermatitis | Dermatitis can be reduced by caloric restriction, various pharmaceuticals and can also disappear without treatment. | Sargent et al., 2015; Hampton et al., 2012; Wilkinson et al., 2020 |
|  | Loss of whiskers | Whisker loss can be caused by overgrooming, whisker eating, stress etc and is reversible. | Sarna et al., 2000 |
|  | Coat condition | Poor coat condition can be treated by adequate companionship, exercise and caloric restriction. | Wilkinson et al., 2020 |
| **Physical/**  **Musculoskeletal** | Tumours | Sodium-glucose cotransporter 2 inhibitors slow tumor growth in mice. | Nasiri et al., 2019 |
|  | Distended abdomen | Can reverse if underlying causes are addressed/modified, such as reduced adiposity and/or less fluid accumulation. | Wilkinson et al., 2020 |
|  | Kyphosis | Voluntary exercise reduces kyphosis. | Ross et al., 2019 |
|  | Tail stiffening | Tail stiffness can be reduced by caloric restriction. | Sell & Monnier, 1997 |
|  | Gait disorders | Sarcopenia is reduced by sodium-glucose cotransporter 2 inhibitors. | Sasaki et al., 2019 |
|  | Tremor | Tremor in mouse models can be treated with many different pharmacological agents (e.g. beta blockers, benzodiazepines). | Kralic et al., 2005 |
|  | Forelimb  grip strength | Sarcopenia is reduced by sodium-glucose cotransporter 2 inhibitors, rapamycin or caloric restriction. | Sasaki et al., 2019; Orenduff et al., 2022 |
|  | Body condition score | Sarcopenia is reduced by sodium-glucose cotransporter 2 inhibitors, rapamycin or caloric restriction. | Sasaki et al., 2019; Orenduff et al., 2022 |
| **Vestibulocochlear/**  **Auditory** | Vestibular disturbance | Spontaneous hair cell regeneration in the inner ear can occur in mice following gentamicin ototoxicity. | Kawamoto et al., 2009 |
|  | Hearing loss | Late life rapamycin decreases age-related hearing loss in mice. | Altschuler et al., 2021 |
|  | Cataracts | Sterols can bind and stabilize soluble crystallin and reduce the severity of cataracts in mice. | Makley et al., 2015 |
|  | Eye discharge/swelling | Can be treated with antibiotics and/or steroids. | Pettan-Brewer & Treuting, 2011 |
|  | Microphthalmia | No current treatments, but this is an active area of drug discovery for new treatment strategies. | Harding et al., 2021 |
|  | Corneal opacity | Sterols can bind and stabilize soluble crystallin and reduce corneal opacity in mice. | Makley et al., 2015 |
|  | Vision loss | Adeno-associated virus delivery of youth-restoring genes (Oct4, Sox2 and Klf4) to the mouse retina reverses vision loss in aging mice. | Yuancheng et al., 2020 |
|  | Menace reflex | If this is due to vision loss, this can be mitigated by adeno-associated virus delivery of youth-restoring genes (Oct4, Sox2 and Klf4) to the mouse retina. | Yuancheng et al., 2020 |
|  | Nasal discharge | Respiratory infections can be attenuated by antibiotic treatment and maintaining a clean cage environment. | Aiello et al., 2016 |
| **Digestive/**  **Urogenital** | Malocclusions | Oral health is improved by rapamycin in aging mice. Incisor teeth can also be trimmed to correct malocclusions. | Burkholder et al., 2012; An et al., 2020 |
|  | Rectal prolapse | Can be treated pharmacologically with drugs that reduce inflammation and surgically by a veterinarian. | Teixeira et al., 2012; Uchihashi et al., 2015 |
|  | Vaginal/uterine/  penile prolapse | Can potentially be treated surgically by a veterinarian. | Wilkinson et al., 2020 |
|  | Diarrhoea | Can be treated pharmacologically with drugs that reduce inflammation. | Teixeira et al., 2012 |
| **Respiratory** | Breathing rate/depth | Treatment with inhaled resveratrol can slow age-related deleterious changes in the mouse lung. | Navarro et al., 2017 |
| **Discomfort** | Mouse  Grimace Scale | Can be reversed if the cause of discomfort is identified and mitigated. | Wilkinson et al., 2020 |
|  | Piloerection | Can be reversed if the cause of piloerection is identified and mitigated (e.g. if hypothermia, increase nesting materials, ensure cage is not near a draft). | Wilkinson et al., 2020 |
| **Other** | Temperature | Hypothermia can be reversed by increasing companionship, more nesting materials and ensuring the cage is not near a draft. | Wilkinson et al., 2020 |
|  | Weight | Excessive weight gain can be reduced by caloric restriction and extreme weight loss can be treated by serving the mice mashed food. | Wilkinson et al., 2020 |

**REFERENCES for Supplementary Table 1:**

1. Aiello, SE, Moses MA, Allen DG. The Merck veterinary manual, Eleventh Edition, Kenilworth, NJ: Merck & Co., Inc., 2016.
2. Altschuler RA, Kabara L, Martin C, Kanicki A, Stewart CE, Kohrman DC, Dolan DF. Rapamycin Added to Diet in Late Mid-Life Delays Age-Related Hearing Loss in UMHET4 Mice. Front Cell Neurosci. 2021 Apr 7;15:658972. doi: 10.3389/fncel.2021.658972.
3. An JY, Kerns KA, Ouellette A, Robinson L, Morris HD, Kaczorowski C, Park SI, Mekvanich T, Kang A, McLean JS, Cox TC, Kaeberlein M. Rapamycin rejuvenates oral health in aging mice. Elife. 2020 Apr 28;9:e54318. doi: 10.7554/eLife.54318.
4. Burkholder T, Foltz C, Karlsson E, Linton CG, Smith JM. Health Evaluation of Experimental Laboratory Mice. Curr Protoc Mouse Biol. 2012;2:145-165. doi:10.1002/9780470942390.mo110217
5. Hampton AL, Hish GA, Aslam MN, Rothman ED, Bergin IL, Patterson KA, Naik M, Paruchuri T, Varani J, Rush HG. Progression of ulcerative dermatitis lesions in C57BL/6Crl mice and the development of a scoring system for dermatitis lesions. J Am Assoc Lab Anim Sci. 2012;51(5):586-93.
6. Harding P, Toms M, Schiff E, Owen N, Bell S, Lloyd IC, Moosajee M. EPHA2 Segregates with Microphthalmia and Congenital Cataracts in Two Unrelated Families. Int J Mol Sci. 2021 Feb 22;22(4):2190. doi: 10.3390/ijms22042190.
7. Kawamoto K, Izumikawa M, Beyer LA, Atkin GM, Raphael Y. Spontaneous hair cell regeneration in the mouse utricle following gentamicin ototoxicity. Hear Res. 2009 Jan;247(1):17-26. doi: 10.1016/j.heares.2008.08.010.
8. Kralic JE, Criswell HE, Osterman JL, O'Buckley TK, Wilkie ME, Matthews DB, Hamre K, Breese GR, Homanics GE, Morrow AL. Genetic essential tremor in gamma-aminobutyric acidA receptor alpha1 subunit knockout mice. J Clin Invest. 2005 Mar;115(3):774-9. doi: 10.1172/JCI23625.
9. Makley LN, McMenimen KA, DeVree BT, Goldman JW, McGlasson BN, Rajagopal P, Dunyak BM, McQuade TJ, Thompson AD, Sunahara R, Klevit RE, Andley UP, Gestwicki JE. Pharmacological chaperone for α-crystallin partially restores transparency in cataract models. Science. 2015 Nov 6;350(6261):674-7. doi: 10.1126/science.aac9145.
10. Nasiri AR, Rodrigues MR, Li Z, Leitner BP, Perry RJ. SGLT2 inhibition slows tumor growth in mice by reversing hyperinsulinemia. Cancer Metab. 2019 Dec 11;7:10. doi: 10.1186/s40170-019-0203-1
11. Navarro S, Reddy R, Lee J, Warburton D, Driscoll B. Inhaled resveratrol treatments slow ageing-related degenerative changes in mouse lung. Thorax. 2017 May;72(5):451-459. doi: 10.1136/thoraxjnl-2016-208964.
12. Orenduff MC, Coleman MF, Glenny EM, Huffman KM, Rezeli ET, Bareja A, Pieper CF, Kraus VB, Hursting SD. Differential effects of calorie restriction and rapamycin on age-related molecular and functional changes in skeletal muscle. Exp Gerontol. 2022 May 24:111841. doi: 10.1016/j.exger.2022.111841.
13. Pettan-Brewer C, Treuting PM. Practical pathology of aging mice. Pathobiol Aging Age Relat Dis. 2011;1. doi: 10.3402/pba.v1i0.7202.
14. Ross JM, Coppotelli G, Branca RM, et al. Voluntary exercise normalizes the proteomic landscape in muscle and brain and improves the phenotype of progeroid mice. *Aging Cell*. 2019;18(6):e13029. doi:10.1111/acel.13029
15. Sargent JL, Koewler NJ, Diggs HE. Systematic Literature Review of Risk Factors and Treatments for Ulcerative Dermatitis in C57BL/6 Mice. Comp Med. 2015 Dec;65(6):465-72.
16. Sarna JR, Dyck RH, Whishaw IQ. The Dalila effect: C57BL6 mice barber whiskers by plucking. Behav Brain Res. 2000 Feb;108(1):39-45. doi: 10.1016/s0166-4328(99)00137-0.
17. Sasaki T. Sarcopenia, frailty circle and treatment with sodium-glucose cotransporter 2 inhibitors. J Diabetes Investig. 2019 Mar;10(2):193-195. doi: 10.1111/jdi.12966.
18. Sell DR, Monnier VM. Age-related association of tail tendon break time with tissue pentosidine in DBA/2 vs C57BL/6 mice: the effect of dietary restriction. J Gerontol A Biol Sci Med Sci. 1997 Sep;52(5):B277-84. doi: 10.1093/gerona/52a.5.b277.
19. Teixeira LB, Epifânio VL, Lachat JJ, Foss NT, Coutinho-Netto J. Oral treatment with Hev b 13 ameliorates experimental colitis in mice. Clin Exp Immunol. 2012 Jul;169(1):27-32. doi: 10.1111/j.1365-2249.2012.04589.x.
20. Uchihashi M, Wilding LA, Nowland MH. Surgical Correction of Rectal Prolapse in Laboratory Mice (Mus musculus). J Am Assoc Lab Anim Sci. 2015 Jul;54(4):433-8.
21. Wilkinson MJ, Selman C, McLaughlin L, et al. Progressing the care, husbandry and management of ageing mice used in scientific studies. Lab Anim. 2020;54(3):225-238. doi:10.1177/0023677219865291
22. Yuancheng Lu, Benedikt Brommer, Xiao Tian, Anitha Krishnan, Margarita Meer, Chen Wang, Daniel L. Vera, Qiurui Zeng, Doudou Yu, Michael S. Bonkowski, Jae-Hyun Yang, Songlin Zhou, Emma M. Hoffmann, Margarete M. Karg, Michael B. Schultz, Alice E. Kane, Noah Davidsohn, Ekaterina Korobkina, Karolina Chwalek, Luis A. Rajman, George M. Church, Konrad Hochedlinger, Vadim N. Gladyshev, Steve Horvath, Morgan E. Levine, Meredith S. Gregory-Ksander, Bruce R. Ksander, Zhigang He, David A. Sinclair. Reprogramming to recover youthful epigenetic information and restore vision. Nature, 2020; 588 (7836): 124 DOI: 10.1038/s41586-020-2975-4.
23. Zhang B, Ma S, Rachmin I, He M, Baral P, Choi S, Gonçalves WA, Shwartz Y, Fast EM, Su Y, Zon LI, Regev A, Buenrostro JD, Cunha TM, Chiu IM, Fisher DE, Hsu YC. Hyperactivation of sympathetic nerves drives depletion of melanocyte stem cells. Nature. 2020 Jan;577(7792):676-681. doi: 10.1038/s41586-020-1935-3.
